# Differential Response of *Acidobacteria* Subgroups to Forest-to-Pasture Conversion and Their Biogeographic Patterns in the Western Brazilian Amazon

**DOI:** 10.3389/fmicb.2015.01443

**Published:** 2015-12-22

**Authors:** Acacio A. Navarrete, Andressa M. Venturini, Kyle M. Meyer, Ann M. Klein, James M. Tiedje, Brendan J. M. Bohannan, Klaus Nüsslein, Siu M. Tsai, Jorge L. M. Rodrigues

**Affiliations:** ^1^Cell and Molecular Biology Laboratory, Center for Nuclear Energy in Agriculture, University of São PauloPiracicaba, Brazil; ^2^Department of Biology, Institute of Ecology and Evolution, University of OregonEugene, OR, USA; ^3^Department of Plant, Soil and Microbial Sciences, Center for Microbial Ecology, Michigan State UniversityEast Lansing, MI, USA; ^4^Department of Microbiology, University of MassachusettsAmherst, MA, USA; ^5^Department of Land, Air and Water Resources, University of California, DavisDavis, CA, USA

**Keywords:** tropical rainforest, land-use change, spatial scale, 16S rRNA gene, community similarity, *Acidobacteria*

## Abstract

Members of the phylum *Acidobacteria* are among the most abundant soil bacteria on Earth, but little is known about their response to environmental changes. We asked how the relative abundance and biogeographic patterning of this phylum and its subgroups responded to forest-to-pasture conversion in soils of the western Brazilian Amazon. Pyrosequencing of 16S rRNA genes was employed to assess the abundance and composition of the *Acidobacteria* community across 54 soil samples taken using a spatially nested sampling scheme at the landscape level. Numerically, *Acidobacteria* represented 20% of the total bacterial community in forest soils and 11% in pasture soils. Overall, 15 different *Acidobacteria* subgroups of the current 26 subgroups were detected, with *Acidobacteria* subgroups 1, 3, 5, and 6 accounting together for 87% of the total *Acidobacteria* community in forest soils and 75% in pasture soils. Concomitant with changes in soil chemistry after forest-to-pasture conversion—particularly an increase in properties linked to soil acidity and nutrient availability—we observed an increase in the relative abundances of *Acidobacteria* subgroups 4, 10, 17, and 18, and a decrease in the relative abundances of other *Acidobacteria* subgroups in pasture relative to forest soils. The composition of the total *Acidobacteria* community as well as the most abundant *Acidobacteria* subgroups (1, 3, 5, and 6) was significantly more similar in composition across space in pasture soils than in forest soils. These results suggest that preponderant responses of *Acidobacteria* subgroups, especially subgroups 1, 3, 4, 5, and 6, to forest-to-pasture conversion effects in soils could be used to define management-indicators of agricultural practices in the Amazon Basin. These acidobacterial responses are at least in part through alterations on acidity- and nutrient-related properties of the Amazon soils.

## Introduction

Land use change driven by human activities is considered the most important factor for biodiversity losses in the tropics (Sala et al., 2000) and a large number of studies have documented the negative effects of land use change for plants, animals (Gibson et al., 2011; Wearn et al., 2012), and most recently, microorganisms (Cenciani et al., 2009; Jesus et al., 2009; Navarrete et al., 2010, 2011, 2013, 2015; Taketani and Tsai, 2010; Rodrigues et al., 2013; Mirza et al., 2014; Mueller et al., 2014; Paula et al., 2014; Ranjan et al., 2015). For example, Rodrigues et al. (2013) reported that forest-to-pasture conversion resulted in a substantial decrease in the abundance of members of the bacterial phylum *Acidobacteria.*

*Acidobacteria* are among the most common bacteria in soils worldwide, including in Amazon soils (Kim et al., 2007; Jesus et al., 2009; Navarrete et al., 2010, 2013, 2015). The analysis of 16S rRNA gene sequences has demonstrated that acidobacterial abundance within a community may be regulated by soil pH (Fierer et al., 2007; Lauber et al., 2008; Jones et al., 2009; Rousk et al., 2010; Kuramae et al., 2011) and nutrient availability (Zhao et al., 2014). Genomic and physiological traits indicate characteristics that may contribute to *Acidobacteria* survival and growth in soil, such as the presence of membrane transporters and the ability to use carbon sources that span from simple sugars to more complex substrates such as hemicellulose, cellulose, and chitin; the reduction of nitrate, nitrite, and possibly nitric oxide; iron scavenging; and production of antimicrobial compounds (Ward et al., 2009; Rawat et al., 2012). In addition, Greening et al. (2015) proposed that consumption of trace gases such as H_2_ provides a dependable general mechanism for *Acidobacteria* to generate maintenance energy required for long-term survival in soils.

Recently, increased attention has been paid to the response of *Acidobacteria* to environmental changes (George et al., 2009; Naether et al., 2012; Catão et al., 2014). Despite this appreciation for the phylum *Acidobacteria*, little is still known about the differential response at subgroup level to alterations in soil chemical properties and fertility, and how their community similarity change with distance in mosaic landscapes. Navarrete et al. (2013) reported the impact of agricultural management of soybean in Amazon forest soils on the composition of the *Acidobacteria* community, and they revealed that the abundance of *Acidobacteria* subgroups was related to soil chemical properties, which were clearly affected by agricultural management. These findings opened the possibility that subgroups of *Acidobacteria* could be used as management-indicators for the consequences of agricultural practices in the Amazon region.

The present study was designed to assess the *Acidobacteria* subgroup response at different geographic scales in primary forest and pasture soils. Firstly, we hypothesized that different subgroups of *Acidobacteria* respond differently to forest conversion into pastures in Amazon soils. Because of the substantial effects that land use change may have on soil chemical characteristics, we evaluated the differential response of *Acidobacteria* subgroups through the prism of the expected changes in soil chemical properties after forest-to-pasture conversion in the Amazon. In a corollary hypothesis, we tested whether taxonomic similarity of total *Acidobacteria* community and of their most abundant subgroups varies across space in forest and pasture soil samples in the western Brazilian Amazon. To address these hypotheses, we used pyrosequencing of the region V4 of the bacterial 16S rRNA gene to analyze the relative abundance and composition of the *Acidobacteria* community inhabiting soil from primary forests and pastures collected from the Amazon Rainforest Microbial Observatory, a model site representing the current expansive agricultural development of the region. We correlated the relative abundances of *Acidobacteria* at the taxonomic levels phylum and subgroup with soil chemical properties to explore group-specific responses to agricultural conversion. Furthermore, we explored the relationship between group-specific biogeographic patterns and land use change by comparing distance-decay relationship patterns.

## Materials and methods

### Site description and soil sampling

This study was performed at the Fazenda Nova Vida (10°10′5″S and 62°49′27″W), located in the central region of the Brazilian state of Rondônia at the Amazon Rainforest Microbial Observatory (ARMO). Soils are classified as red-yellow podzolic latosol (Kandiudult). The climate is humid tropical, with an annual average temperature of 25.5°C and an average precipitation of 2200 mm (Bastos and Diniz, 1982). Local farmers employ slash-and-burn practices, i.e., clearing of primary forest followed by burning, in order to support livestock and farming systems in this region.

Soil samples were collected at the end of the rainy season (April 2009) from three primary forest sites and three pasture sites that had been continuously managed since 1987. At each site, a nested sampling scheme was established, centered on a 100 × 100 m (100 m^2^) quadrat, with 10 × 10 m (10 m^2^), and 1 × 1 m (1 m^2^) quadrats nested within and adjacent to one corner of the 100 m^2^ quadrat, for a total of nine sampling points per 100 m^2^ quadrat (Figure S1). At each point, after the removal of the litter layer, the soil was sampled from 0 to 10 cm depth in the topsoil layer, gently homogenized, and subdivided. Samples were transported to the laboratory on ice. A portion of each sample was stored at −80°C for molecular analysis and another portion was stored at 4°C for soil chemical analysis.

### Soil chemical properties and statistical analysis

The soil samples were dried and passed through a sieve (149 μm size). Total carbon (C) and nitrogen (N) were measured on a LECO CN elemental analyzer (St. Joseph, MI, USA) at the Soil Biogeochemistry Laboratory, Center for Nuclear Energy in Agriculture, University of São Paulo, Brazil. Soil chemical properties for each sample were analyzed at the Laboratory of Soil Fertility, Luiz de Queiroz College Agriculture, University of São Paulo, Brazil. Soil pH was measured from a soil/water (1:2.5) suspension. Aluminum (Al), calcium (Ca), and magnesium (Mg) were extracted with 1 M potassium chloride. Ca and Mg were determined by atomic absorption spectrometry, while Al was determined by acid-base titration. Phosphorous (P) and potassium (K) were extracted by ion-exchange resin, and determined by colorimetry and atomic emission spectroscopy, respectively. Combined results were used for calculation of exchangeable bases (SB) as the sum of Ca, Mg, and K; cation-exchange capacity (CEC) as the sum of Ca, Mg, K, Al, and H; base saturation (V) as the percent relation between SB and CEC; aluminum saturation (m) as the percent relation between exchangeable Al and CEC; and potential acidity (H+Al), by an equation based on the pH determined in Shoemaker-McLean-Pratt (SMP) buffer solution. Analysis of similarity (ANOSIM) statistics was calculated to test for differences between forest and pasture soil chemical properties. A distance matrix (Euclidean metric) was constructed using non-transformed data. ANOSIM was carried out using Primer six (version 6.1.5, Primer-E Ltd., Plymouth, UK).

### Isolation of DNA from soil, amplification, and pyrosequencing of bacterial 16S rRNA genes

Total genomic DNA for each soil sample was extracted in triplicate using the Power Soil DNA Isolation Kit (Mo Bio Laboratories Inc., Carlsbad, CA, USA), according to the manufacturer's instructions. The extractions for each sample were combined and DNA was quantified spectrophotometrically (Nanodrop ND-1000, NanoDrop Technologies, Inc., Wilmington, DE, USA). All DNA samples were stored at −20°C. The primer set 577F (5′-AYTGGGYDTAAAGNG-3′) and 926R (5′-CCGTCAATTCMTTTRAGT-3′) targeting the V4 region of bacterial 16S rRNA gene was used for the amplification. Group-specific primers for *Acidobacteria* such as Acid31F (Barns et al., 1999) and ACIDO (Lee and Cho, 2011) were not used in order to avoid the selective amplification and not detection of members of the phylum *Acidobacteria* such as 2, 22, and 25 as reported in many studies (Sait et al., 2006; Barns et al., 2007; George et al., 2009; Jones et al., 2009; Kielak et al., 2009; Lee and Cho, 2011). Adapter sequence was added to the primers as recommended by Roche (Table S1). Barcodes of 8 bp and AC linker were added to forward primers only. Each reaction was carried out in 50 μl reactions containing 1 × buffer, 1.8 mM of MgCl_2_, 0.2 μM of each primer, 200 μM of deoxynucleoside triphosphate, 300 ng/μl of bovine serum albumin, 10 ng of DNA template and 1 μl of the enzyme FastStart High Fidelity PCR System (Roche Applied Sciences, Indianapolis, IN, USA), subjected to the following conditions: 95°C for 3 min; 30 cycles of 94°C for 45 s, 57°C for 45 s and 72°C for 1 min; and 72°C for 4 min. Each soil sample was amplified in triplicate, and reaction products were pooled and purified using the Qiagen PCR purification kit (Qiagen, Valencia, CA, USA). PCR products were sequenced on a 454 GS FLX Sequencer (454 Life Sciences, Branford, CT, USA) at the Michigan State University Research Technology Support Facility. To prevent the possibility of sequencing errors (Huse et al., 2007), all reads were removed that either contained one or more ambiguous bases (N), had lengths outside the main distribution, or presented inexact matches to the primers used in the study. The high-quality bacterial 16S rRNA gene sequences are available through FigShare, http://dx.doi.org/10.6084/m9.figshare.1547935.

### Sequence analysis and statistics

Sequences were processed using the bioinformatics platform QIIME version 1.7 (Caporaso et al., 2010). Sequences were removed from the analysis if they did not have the primer sequence, were less than 300 nt or more than 400 nt in length, contained a homopolymer run exceeding twenty nucleotides, or had ambiguous characters. The remaining sequences were assigned to samples by matching them to barcode sequences. Sequences that passed these quality filters were clustered into OTUs with a similarity cutoff of 97% using UCLUST (Edgar, 2010). Taxonomy was assigned to representative sequences from each OTU using the Ribosomal Database Project (Wang et al., 2007) web-based taxonomy assignment tool (http://rdp.cme.msu.edu/index.jsp) version 2.6 against the RDP 16S rRNA training set 9. The OTU table was filtered for specific taxonomic groups, and the relative abundance of *Acidobacteria* was estimated by comparing the number of sequences classified as belonging to the phylum with the number of classified bacterial sequences in each sample. Similarly, the relative abundance of *Acidobacteria* subgroups was estimated across all individual samples by comparing the number of sequences classified as belonging to each subgroup with the number of classified *Acidobacteria* sequences. Explicit relationships between the relative abundance of *Acidobacteria* subgroups and soil chemical properties were examined using constrained ordination generated by redundancy analysis (RDA) with the software CANOCO 4.5 (ter Braak and Šmilauer, 2002). Spearman's rank correlation coefficients were calculated between the relative abundance of *Acidobacteria* subgroups and soil properties using the “multtest” package (Pollard et al., 2005) in R (R Core Team, 2015). *P*-values were corrected for multiple testing, using the false discovery rate controlling procedure (Benjamini and Hochberg, 1995).

### Distance-decay of similarity analyses

The pairwise geographic distances between cores were calculated based on geographic coordinates and physical measurements. Community turnover (i.e., the distance-decay of similarity) was determined by regressing the pairwise community similarity against the pairwise logarithm of geographic distance using linear regression. Distance-decay slopes within taxonomic groups were compared between land types using the function diffslope in the software package “simba” (Jurasinski and Retzer, 2012) in R (R Core Team, 2015).

## Results

### Soil chemical properties

Overall, statistical comparison of soil chemical properties for forest and pasture soils indicated that forest conversion to pasture resulted in an increase in properties linked to soil acidity and nutrient availability in soil (Table S2). The chemical composition (Table S2) of forest and pasture soils differed significantly (ANOSIM, *R* = 0.680, *P* = 0.002). Potential acidity (H+Al) was significantly lower in forest soils compared to the pasture soils. Forest soils had significantly lower total C, N, S, and Mg contents and C/N ratios than pasture soils (Table S2).

### Links between the phylum *Acidobacteria*, relative abundances of subgroup-levels, and soil chemical properties

**Figure 1:**
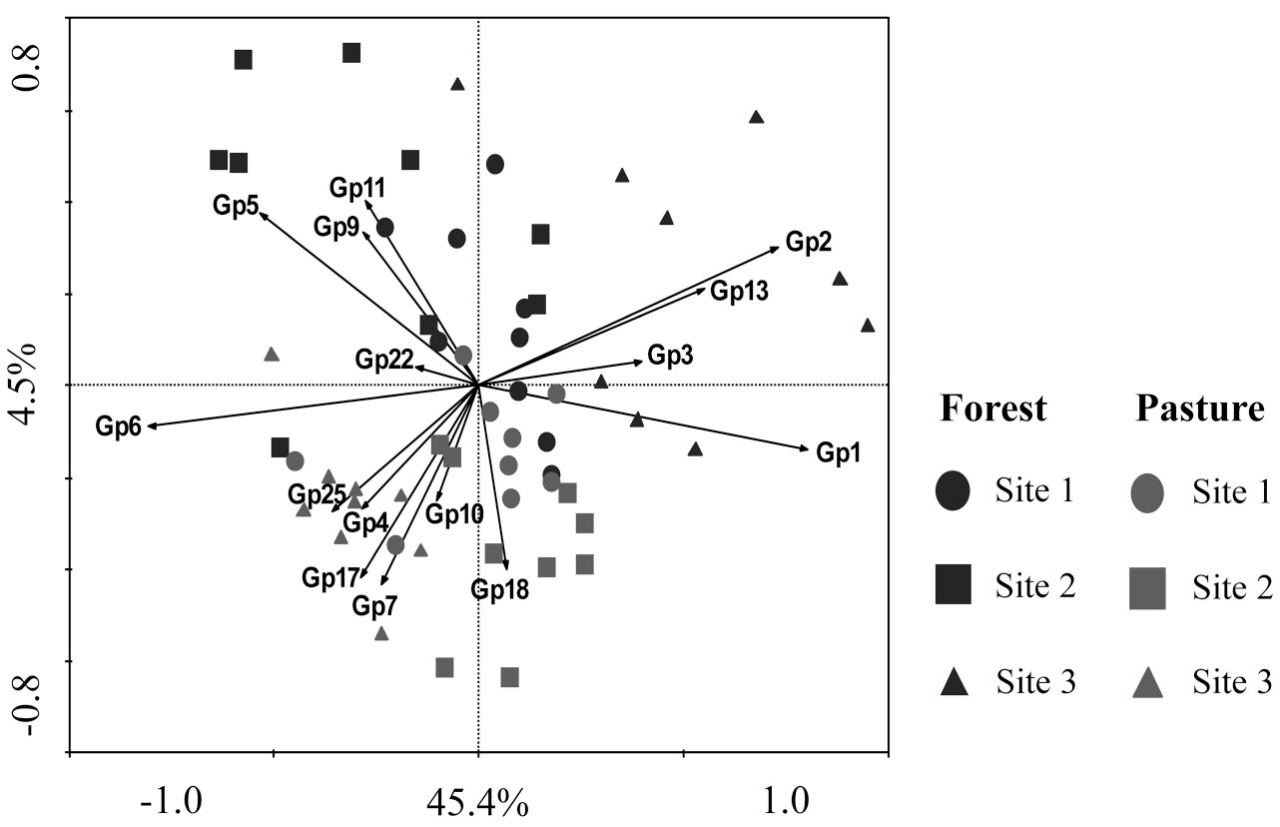
**Constrained ordination diagram for sample plots in the first two redundancy analysis (RDA) axes based on the soil chemical characteristics of the different sampling sites and their relationship with the relative abundance of ***Acidobacteria*** subgroups (1–7, 9–11, 13, 17, 18, 22, and 25)**. Each vector points to the direction of increase for a given *Acidobacteria* subgroup (Gp) and its length indicates the strength of the correlation between this variable and the ordination scores.

The taxonomic analysis of the soil acidobacterial community was based on the retrieval of approximately 45,000 and 20,000 sequences of acidobacterial 16S rRNA gene fragments from forest soils and pasture soils, respectively (Table S3). The relative abundance of *Acidobacteria* sequences within an individual soil bacterial community represented on average 20% (±3.5%) in forest soil samples and 11% (±3.3%) in pasture soil samples. Overall, 15 different *Acidobacteria* subgroups of the current 26 subgroups (Hugenholtz et al., 1998; Zimmermann et al., 2005; Barns et al., 2007) were detected across the 54 soil samples, with *Acidobacteria* subgroups 1, 3, 5, and 6 accounting together for 87% of the total *Acidobacteria* community in forest soils and 75% in pasture soils (Table 1). A redundancy analysis of the relative abundance of *Acidobacteria* subgroups (1–7, 9–11, 13, 17, 18, 22, and 25) showed that the subgroups 1–3, 5, 9, 11, and 13 were significantly associated with forest soils while subgroups 4, 7, 10, 17, 18, and 25 were associated with pasture soils (Figure 1). *Acidobacteria* subgroup 6 was more related to pasture soils than forest soils. Statistically significant differences between forest vs. pasture soils were found for the relative abundances of the *Acidobacteria* subgroups 2 (*P* < 0.005), 4 (*P* < 0.05), 7 (*P* < 0.0005), 10 (*P* < 0.05), 13 (*P* < 0.0005), 17 (*P* < 0.0005) and 18 (*P* < 0.005) (Table 1). A correlation between the relative abundances of *Acidobacteria* subgroups and soil chemical properties revealed two distinct groups. *Acidobacteria* subgroups 1, 2, 3, and 13 were negatively correlated with total C and N content, C/N ratio, and P, S, K, Ca, and Mg content, and positively correlated with properties linked to soil acidity such as pH, Al, H+Al, and m; while subgroups 4, 5, 6, 7, 17, and 25 were positively correlated to nutrient availability and negatively correlated to properties linked to soil acidity (Table 2).

**Table 1:** **Percentage of ***Acidobacteria*** subgroups relative to all ***Acidobacteria*** and of these to all ***Bacteria*** in forest and pasture sites**.

	**Forest sites**			**Pasture sites**			**Statistics**
	**F1**	**F2**	**F3**	**P1**	**P2**	**P3**	**F vs. P**
Gp1	21.30 (11.6–28.5)^a^	9.67 (3.0–17.0)	32.76 (15.1–43.0)	13.02 (8.0–23.6)	26.17 (19.3–40.7)	12.34 (6.7–16.7)	ns^c^
Gp2	2.05 (0–4.4)	0.93 (0.3–2.0)	11.62 (2.4–22.3)	0.86 (0.5–2.0)	1.34 (0–3.6)	0.61 (0–1.4)	^**^
Gp3	29.31 (23.4–33.5)	15.80 (10.2–21.7)	24.73 (19.9–29.8)	16.8 (11.8–31.3)	19.06 (2.8–29.2)	19.59 (11.0–23.7)	ns
Gp4	3.18 (0–6.0)	5.31 (2.3–7.5)	1.67 (0.2–7.8)	4.29 (1.8–21.0)	4.76 (0.2–8.9)	12.55 (3.4–54.6)	^*^
Gp5	14.5 (9.6–18.6)	22.04 (16.7–30.8)	8.32 (4.8–14.5)	10.42 (8.9–26.5)	4.52 (1.7–9.0)	15.53 (8.9–24.9)	ns
Gp6	24.83 (16.8–33.7)	40.46 (30.1–51.6)	16.9 (4.6–35.4)	17.15 (19.7–37.4)	31.96 (20.4–44.0)	38.37 (27.8–45.7)	ns
Gp7	1.27 (0–3.0)	1.96 (0.2–3.8)	0.72 (0–1.7)	1.31 (0.6–4.0)	3.81 (2.0–5.5)	2.71 (1.8–5.0)	^***^
Gp9	0.02 (0–0.1)	0.31 (0–1.9)	ND^b^	0.01 (0–0.07)	ND	ND	ns
Gp10	0.06 (0.1–0.2)	0.12 (0–0.5)	0.13 (0.1–0.3)	0.1 (0–0.5)	0.23 (0–0.6)	0.25 (0–0.9)	^*^
Gp11	0.09 (0.2–0.5)	0.29 (0–1.2)	ND	0.01 (0–0.3)	0.01 (0–0.1)	0.04 (0–0.2)	ns
Gp13	1.80 (0.2–7.0)	0.55 (0–1.0)	2.3 (0.8–4.8)	0.34 (0–0.8)	0.49 (0–1.3)	0.26 (0–0.5)	^***^
Gp17	0.66 (0.3–1.8)	0.06 (0.03–1.9)	0.27 (0–0.3)	1.7 (1.3–5.4)	2.54 (0–3.6)	1.71 (1.2–2.7)	^***^
Gp18	0.05 (0.1–0.2)	0.05 (0–0.2)	0.01 (0–0.1)	0.13 (0–0.3)	0.43 (0–1.0)	0.08 (0–0.3)	^**^
Gp22	0.08 (0.2–0.3)	0.2 (0–1.0)	0.01 (0–0.05)	0.02 (0–0.3)	0.04 (0–0.2)	0.13 (0–0.3)	ns
Gp25	0.34 (0.2–0.6)	0.86 (0.4–1.4)	0,.05 (0–0.1)	0.05 (0–0.8)	0.97 (0–2.4)	0.93 (0.2–1.5	ns
unclassified *Acidobacteria*	0.23 (0.3–0.9)	0.36 (0–0.8)	0.2 (0–0.4)	0.09 (0–0.3)	0.04 (0–0.2)	0.28 (0–0.6)	ns
Total *Acidobacteria* community	17.51 (13.5–23.0)	20.1 (14.0–29.7)	24.15 (13.3–35.3)	7.26 (7.7–21.1)	14.3 (10.1–19.6)	11.08 (6.6–14.4)	ns

a Average and range (%) of the average for each of nine replicate soils in each site.

b ND indicates that sequences of this subgroup were not detected. DNA sequences were classified into 26 acidobacterial subgroups using the Ribosomal Database Project 2 classifier (release 10.4). The 26 subgroups are classified according to the following designations: subgroups 1–8 according to Hugenholtz et al. (1998); subgroups 9–11 according to Zimmermann et al. (2005), and subgroups 12–26 according to Barns et al. (2007).

c Tukey's honestly significant difference (HSD) test was performed considering all pairwise comparisons between the 27 soil cores for forest sites and 27 soil cores for pasture sites. Significance levels: ns: P > 0.05,

* P < 0.05,

** P < 0.005,

*** P < 0.0005.

**Table 2:** **Spearman's rank correlation coefficients and statistical significance between abundance of ***Acidobacteria*** subgroups relative to all ***Acidobacteria*** and soil properties**.

**Soil properties**	***Acidobacteria*** **subgroups**														
	**Gp1**	**Gp2**	**Gp3**	**Gp4**	**Gp5**	**Gp6**	**Gp7**	**Gp9**	**Gp10**	**Gp11**	**Gp13**	**Gp17**	**Gp18**	**Gp22**	**Gp25**
pH	−0.535^***^	−0.419^**^	−0.302^*^	0.396^**^		0.529^***^					−0.398^**^				0.281^*^
N	−0.438^***^	−0.648^***^	−0.428^***^	0.571^***^		0.494^***^	0.455^***^				−0.599^***^	0.581^***^	0.307^*^		0.307^*^
C	−0.414^***^	−0.607^***^	−0.453^***^	0.549^***^		0.507^***^	0.515^***^				−0.611^***^	0.598^***^	0.362^**^		0.314^*^
C/N					−0.331^**^									−0.277^*^	
P	−0.678^***^	−0.446^***^	−0.256^*^	0.441^***^	0.455^***^	0.613^***^					−0.400^**^	0.263^*^			
S		−0.335^*^		0.290^*^							−0.294^*^	0.262^*^			0.367^**^
K	−0.514^***^	−0.324^*^		0.367^**^	0.303^*^	0.522^***^								0.265^*^	
Ca	−0.615^***^	−0.551^***^	−0.292^*^	0.570^***^	0.535^***^	0.518^***^		0.450^***^		0.291^*^	−0.465^***^	0.262^*^			0.271^*^
Mg	−0.393^***^	−0.494^***^		0.474^***^	0.290^*^	0.364^**^					−0.425^***^	0.354^**^			0.336^*^
Al	0.574^***^	0.478^***^		−0.431^***^	−0.400^**^	−0.496^***^		−0.300^*^	0.279^*^	−0.353^**^	0.448^***^				
H+Al	0.281^*^						0.312^*^						0.296^*^		
CEC	−0.390^***^	−0.540^***^	−0.414^**^	0.409^**^	0.310^*^	0.425^***^		0.365^**^			−0.422^***^	0.258^*^	0.256^*^		0.417^**^
V	−0.649^***^	−0.445^***^	0.544^***^		0.518^***^	0.511^***^		0.400^**^		0.316^*^	−0.363^**^				
m	0.644^***^	0.576^***^		−0.596^***^	−0.505^***^	−0.551^***^		−0.436^***^		−0.351^*^	0.449^***^	−0.280^*^			−0.311^*^

Significance levels for the Spearman's rank coefficients are indicated at the

* P < 0.05,

** P < 0.005,

*** *P < 0.0005 levels. H+Al, potential acidity; CEC, cation exchange capacity; V, base saturation index; m, Al saturation index. Reference units are explained in Supplementary Table S2*.

### Acidobacterial distance-decay relationships

**Figure 2:**
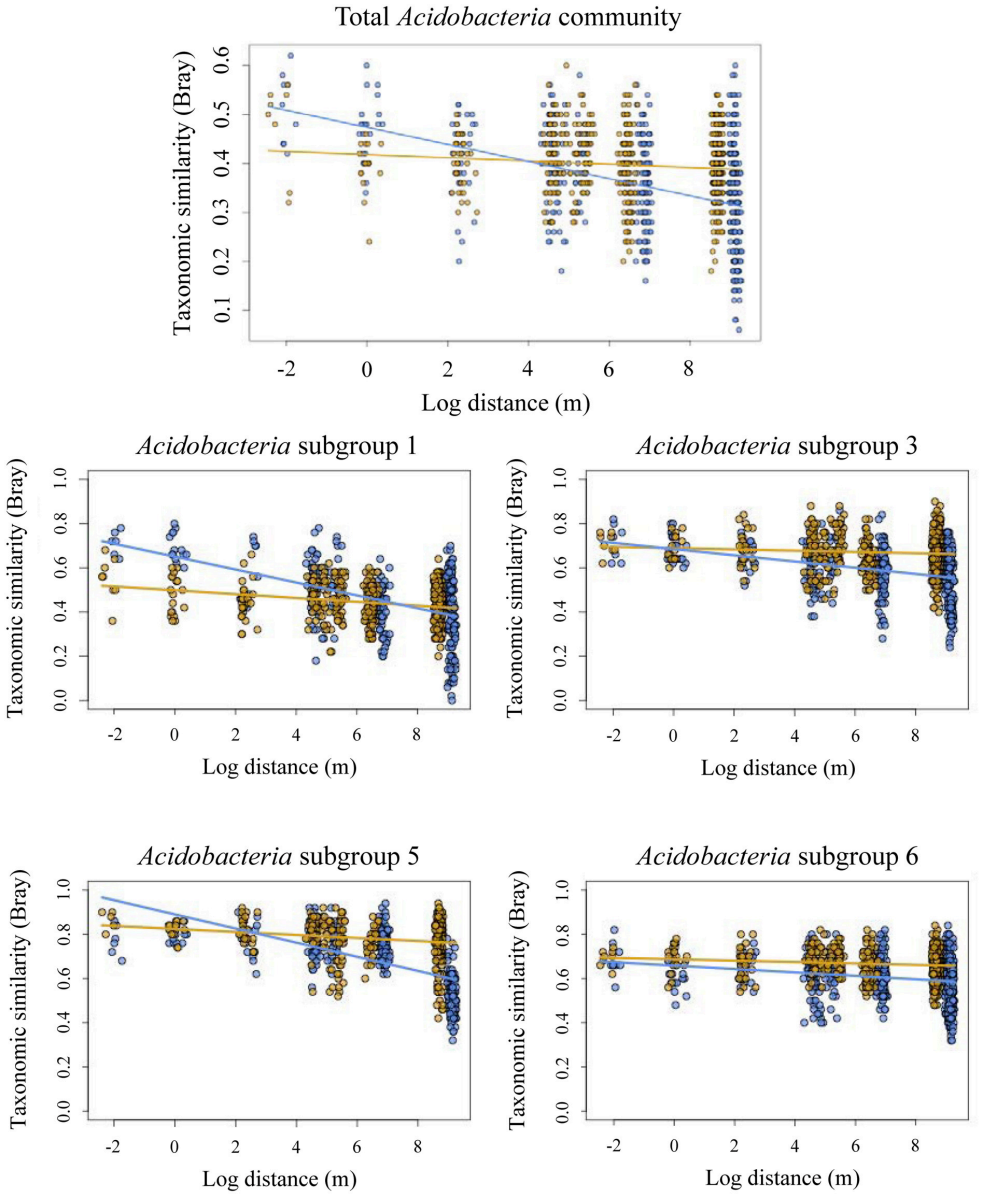
**Decay of taxonomic similarity (Bray–Curtis) with geographic distance in forest (blue) and pasture (yellow) for total acidobacterial community and ***Acidobacteria*** subgroups 1, 3, 5, and 6**.

Taxonomic similarity of the total *Acidobacteria* community was significantly correlated with geographic distance in both forest and pasture sites (Table 3). The slopes of the lines fitted to these relationships differed significantly between the forest and pasture soils with a significantly steeper slope for the total forest *Acidobacteria* community (Figure 2).

**Table 3:** **Correlations of taxonomic similarity (Bray Curtis) and geographic distance of phylum ***Acidobacteria*** and subgroups with comparison of slope of linear model between land use types**.

	**Forest mantel R**	**Pasture mantel R**	**Difference in slope**
Total *Acidobacteria* community	0.4133^***^	0.1369^**^	−0.01419^***^
Gp1	0.454^***^	0.255^***^	−0.02049^***^
Gp3	0.3074^***^	0.096^*^	−0.01125^***^
Gp5	0.636^***^	0.198^***^	−0.02518^***^
Gp6	0.1835^**^	0.1462^**^	−0.004582^**^

Significance levels:

* P < 0.05,

** P < 0.01,

*** P < 0.001.

Taxonomic similarity was significantly correlated with distance for *Acidobacteria* subgroups 1, 3, 5, and 6 in both the forest and pasture soils (Table 3). For each group, forest distance-decay slopes were significantly steeper than their pasture counterparts (Figure 2). The distance-decay linear model showed a better fit to community similarity over distance for forest *Acidobacteria* communities than for those from pasture. Similar biogeographic patterns were revealed for the total *Acidobacteria* community and total bacterial community when comparing slopes across all forest and pasture soils (Figure S2).

## Discussion

The present study reports differential relative abundances for *Acidobacteria* at phylum and subgroup-levels in forest soils and in soils converted into pasture in the western Brazilian Amazon. These differences in abundances are correlated with soil acidity and nutrient availability. Total *Acidobacteria* community as well as the most abundant subgroups, namely 1, 3, 5, and 6, showed a divergence in spatial patterning between forest and pasture, with the pasture communities showing less spatial turnover than the forest communities.

Pasture establishment on acidic soils in the Amazon region is preceded by cutting and removing the economically important trees and burning the remaining above ground biomass (Fujisaka et al., 1996). As a result of these conversion and management practices, the thick organic layer of the forest is lost, the soil nutrient input is changed, and the topsoil is fertilized with alkaline ashes, thus increasing the soil pH (Juo and Manu, 1996; Giardina et al., 2000; Makeschin et al., 2008). Neye and Greenland (1960) proposed the “nutrient-rich ash” hypothesis to explain the observed short-term increase in soil nutrient availability after slash-and-burn clearing of forest. Although the slash-and-burn method of deforestation was applied 28 years before the soil sampling in our pasture sites, numerous studies of forest-to-pasture conversion in the Amazon reported increases in C and N stocks after several years of pasture establishment (Feigl et al., 1995; Neill et al., 1995, 1996; Cerri et al., 2004). Increases in C and N contents and nutrient availability in pasture soils can be also associated with a more decomposable litter (Rhoades et al., 2000; Potthast et al., 2010) and a dense fine-root system (Rhoades and Coleman, 1999) of the pasture grasses.

The chemical characteristics found in pasture soils can be a selective pressure for soil bacteria that prefer nutrient-rich habitats. Cultivation-dependent and -independent approaches have revealed adaptations of members of the phylum *Acidobacteria* to low substrate concentrations in soil, and their negative responses to increases in carbon and pH (Noll et al., 2005; Eichorst et al., 2007; Fierer et al., 2007; Ward et al., 2009). However, certain subgroups of the *Acidobacteria* are also known to have a preference for soil environments with increased available nutrients, i.e., copiotrophic environments (Navarrete et al., 2013). Despite the higher abundance of most *Acidobacteria* subgroups in forest soils, which may help to explain the strong decrease in the proportion of the total *Acidobacteria* community after forest-to-pasture conversion (Rodrigues et al., 2013), subgroups 7, 17, and 18 were significantly more abundant in pasture soils compared to the forest soils, with their abundances linked to high nutrient availability. *Acidobacteria* subgroup 7 showed similar response in soils from the Southeastern Brazilian Amazon converted into agricultural fields, with their abundances linked to high contents of nutrient in soil (Navarrete et al., 2013). Naether et al. (2012) also found higher relative abundances for members of *Acidobacteria* subgroups 17 in pasture soils in comparison to forest soils from three geographical regions in Germany. The selective advantage that allows microorganisms to respond rapidly in environments characterized by fluctuations in resource availability may be conferred by the number of rRNA gene copies in their genomes (Klappenbach et al., 2000; Stevenson and Schmidt, 2004). Genomes of *Acidobacteria* subgroups 1 and 3 were typified by a low number of rRNA gene copies (Ward et al., 2009). Although the number of rRNA gene copies is unknown for most of the *Acidobacteria* subgroups, the few number of ribossomal operons in acidobacterial genomes (Ward et al., 2009) is consistent with the higher abundance of this phylum in forest soils and has been postulated to be a characteristic marker of slow growth and a *K*-selected lifestyle (Klappenbach et al., 2000; Stevenson and Schmidt, 2004). Taken together, these findings suggest that different *Acidobacteria* subgroups have different life history patterns, with some preferring high nutrient concentrations and others preferring more oligotrophic environments.

The *Acidobacteria* subgroups 4 and 10 were also predominant in pasture soils and positively linked to soil pH. Previously, the abundance of the *Acidobacteria* subgroup 4 has been linked to increases in soil pH (Jones et al., 2009; Lauber et al., 2009). In addition, *Blastocatella fastidiosa*, the only known isolate from *Acidobacteria* subgroup 4, recovered from a savanna soil with a moderate acidic pH (i.e., close to 6.0) in Namibia, grows at even higher pHs (up to 10.0) (Foesel et al., 2013). Although soil pH has been demonstrated to explain a significant degree of microbial community variation in different spatial scales (Lauber et al., 2009), few studies have characterized the specific effects of pH on rare *Acidobacteria* subgroups in soil.

A large fraction of the total *Acidobacteria* community was composed of members of subgroup 1 in both forest and pasture soils. Sait et al. (2006) identified moderately acidic pH values as an important factor driving the abundance of members of this *Acidobacteria* subgroup in different soils, with *Acidobacteria* subgroup 1 increasing in relative abundance as the soil pH decreases. Li et al. (2014) showed significant negative correlations between *Acidobacteria* subgroup 1 and pH, and a positive correlation with C/N ratio. Rawat et al. (2013, 2014) and Ward et al. (2009) reported that members of *Acidobacteria* subgroup 1 are versatile heterotrophs that hydrolyze a suite of sugars and complex polysaccharides, contributing to carbon availability in certain ecosystems, including oligotrophic environments. This consideration was based on genomic data from *Granulicella mallensis* MP5ACTX8^T^ and *Granulicella tundricola* type strain MP5ACTX9^T^, members of *Acidobacteria* subgroup 1 from tundra soil, and two acidobacterial subgroup 1 strains (*Acidobacterium capsulatum*), isolated from sediments in acidic drainage from the Yanahara pyrite mine in Japan. Isolation, cultivation and genome analysis of *Acidobacteria* subgroup 1 community members has revealed sugars as their preferred growth substrates (Männistö et al., 2011), and metabolic versatility with genes involved in metabolism and transport of carbohydrates, utilization and biosynthesis of diverse structural and storage polysaccharides such as plant based carbon polymers (Rawat et al., 2014).

The spatial turnover of a community (i.e., the rate of the distance-decay relationship) has been used as a proxy to estimate biotic homogenization at the landscape scale (Olden and Poff, 2003; Rodrigues et al., 2013). Through our approach we were able to detect changes to the spatial patterning of the *Acidobacteria* community as well as the most abundant *Acidobacteria* subgroups. In all cases, the directionality of change was the same; forest communities showed a steeper distance-decay relationship relative to pasture communities and that pasture communities were more similar to each other at larger distances than forest communities. We take these patterns to be suggestive of biotic homogenization. Changes to distance-decay patterns could result from alterations to several community assembly processes. For example, forest soils may have a more diverse or spatially variable array of microbial niches that may get broken down through the change in aboveground plant communities or alterations to the soil environment associated with land use change. It has been shown that *Acidobacteria* are one of the most abundant members of the phyllosphere of tropical trees, and that the distribution of *Acidobacteria* follows host plant phylogeny (Kim et al., 2012). Hence the removal and subsequent replacement of the tree community by low diversity grassland could be a strong driver in the changes to *Acidobacteria* biogeography.

These differential responses in relative abundance and biogeographic patterning of the *Acidobacteria* phylum and its subgroups to forest conversion into pastures in the Amazon rainforest expand the known possibilities to explore these subgroups to define management-indicators of agricultural practices. When conditions related to specific soil properties change owing to soil management practices, the proportion of different subgroups may be used to as an indicator of the soil status (Holt and Miller, 2011; Kuramae et al., 2011).

In conclusion, this study expands the understanding of ecological characteristics of *Acidobacteria* subgroups in Amazon soils by reporting differential responses of *Acidobacteria* and their subgroups to forest-to-pasture conversion and the associated biogeographic patterns in a western Brazilian Amazon area. The forest clear-cutting and burning in the Amazon primarily to yield cattle pastures play a role in the assembly of the *Acidobacteria* communities in soil, especially in *Acidobacteria* subgroups 1, 3, 4, 5, and 6. Preponderant responses of *Acidobacteria* subgroups to forest-to-pasture conversion effects in soils are at least in part through effects on soil acidity and nutrient availability. The results also showed more similar composition of the total *Acidobacteria* community as well as the most abundant *Acidobacteria* subgroups across space in pasture soils than in forest soils. Taken together, these findings could assist to define management-indicators to judge the impacts from the forest-to-pasture conversion on soil ecosystem in the Amazon Basin.

## Author contributions

AN and JR designed research; AN, AV, KM, AK, JT, KN, BB, ST, and JR performed research; JT, BB, ST, KN, and JR contributed new reagents/analytic tools; AN, AV, KM, AK, and JR analyzed data; and AN, KM, AK, BB, KN, and JR wrote the paper.

### Conflict of interest statement

The authors declare that the research was conducted in the absence of any commercial or financial relationships that could be construed as a potential conflict of interest.

## References

[B1] Barns S. M. Cain E. C. Sommerville L. Kuske C. R. (2007). *Acidobacteria* phylum sequences in uranium-contaminated subsurface sediments greatly expand the known diversity within the phylum. Appl. Environ. Microbiol. 73, 3113–3116. 10.1128/AEM.02012-06 17337544 PMC1892891

[B2] Barns S. M. Takala S. L. Kuske C. R. (1999). Wide distribution and diversity of members of the bacterial kingdom Acidobacterium in the environment. Appl. Environ. Microbiol. 65, 1731–1737. 10103274 10.1128/aem.65.4.1731-1737.1999 PMC91244

[B3] Bastos T. X. Diniz T. D. (1982). Avaliação do Clima do Estado de Rondônia Para Desenvolvimento Agrícola. Belém: Embrapa-CPATU.

[B4] Benjamini Y. Hochberg Y. (1995). Controling the false discovery rate - a practical and powerful approach to multiple testing. J. R. Stat. Soc. B Stat. Methodol. 57, 289–300.

[B5] ter Braak C. J. F. Šmilauer P. (2002). CANOCO Reference Manual and CanoDraw for Windows User's Guide: Software for Canonical Community Ordination (Version 4.5). New York, NY: Microcomputer Power.

[B6] Caporaso J. G. Kuczynski J. Stombaugh J. Bittinger K. Bushman F. D. Costello E. K.. (2010). QIIME allows analysis of high-throughput community sequencing data. Nat. Methods 7, 335–336. 10.1038/nmeth.f.303 20383131 PMC3156573

[B7] Catão E. C. P. Lopes F. A. C. Araújo J. F. Castro A. P. Barreto C. C. Bustamante M. M. C.. (2014). Soil acidobacterial 16S rRNA gene sequences reveal subgroup level differences between savanna-like Cerrado and Atlantic forest brazilian biomes. Int. J. Microbiol. 2014:156341. 10.1155/2014/156341 25309599 PMC4181792

[B8] Cenciani K. Lambais M. R. Cerri C. C. De Azevedo L. C. B. Feigl B. J. (2009). Bacteria diversity and microbial biomass in forest, pasture and fallow soils in the southwestern Amazon Basin. Rev. Bras. Cienc. Solo 33, 907–916. 10.1590/S0100-06832009000400015

[B9] Cerri C. E. P. Paustian K. Bernoux M. Victoria R. L. Melillos J. M. Cerri C. C. (2004). Modeling changes in soil organic matter in Amazon forest to pasture conversion with the Century model. Glob. Change Biol. 10, 815–832. 10.1111/j.1365-2486.2004.00759.x

[B10] Edgar R. C. (2010). Search and clustering orders of magnitude faster than BLAST. Bioinformatics 26, 2460–2461. 10.1093/bioinformatics/btq461 20709691

[B11] Eichorst S. A. Breznak J. A. Schmidt T. M. (2007). Isolation and characterization of soil bacteria that define Terriglobus gen. nov., in the phylum Acidobacteria. Appl. Environ. Microb. 73, 2708–2717. 10.1128/AEM.02140-06 17293520 PMC1855589

[B12] Feigl B. J. Melillo J. M. Cerri C. C. (1995). Changes in the origin and the quality of soil organic matter after pasture introduction in Rondônia (Brazil). Plant Soil 175, 21–29. 10.1007/BF02413007

[B13] Fierer N. Bradford M. A. Jackson R. B. (2007). Toward an ecological classification of soil bacteria. Ecology 88, 135–1364. 10.1890/05-1839 17601128

[B14] Foesel B. U. Rohde M. Overmann J. (2013). Blastocatella fastidiosa gen. nov., sp. nov., isolated from semiarid savanna soil - the first described species of Acidobacteria subdivision 4. Syst. Appl. Microbiol. 36, 82–89. 10.1016/j.syapm.2012.11.002 23266188

[B15] Fujisaka S. Bell W. Thomas N. Hurtad L. Crawford E. (1996). Slash-and-burn agriculture, conversion to pasture, and deforestation in two Brazilian Amazon colonies. Agr. Ecosyst. Environ. 59, 115–130. 10.1016/0167-8809(96)01015-8

[B16] George I. F. Liles M. R. Hartmann M. Ludwig W. Goodman R. M. Agathos S. N. (2009). Changes in soil Acidobacteria communities after 2,4,6-trinitrotoluene contamination. FEMS Microbiol. Lett. 296, 159–166. 10.1111/j.1574-6968.2009.01632.x 19459956

[B17] Giardina C. P. Sanford R. L. Døckersmith I. C. Jaramillo V. J. (2000). The effects of slash burning on ecosystem nutrients during the land preparation phase of shifting cultivation. Plant Soil 220, 247–260. 10.1023/A:1004741125636

[B18] Gibson L. Lee T. M. Koh L. P. Brook B. W. Gardner T. A. Barlow J.. (2011). Primary forests are irreplaceable for sustaining tropical biodiversity. Nature 478, 378–381. 10.1038/nature10425 21918513

[B19] Greening C. Carere C. R. Rushton-Green R. Harold L. K. Hards K. Taylor M. C.. (2015). Persistence of the dominant soil phylum *Acidobacteria* by trace gas scavenging. Proc. Natl. Acad. Sci. U.S.A. 112, 10497–10502. 10.1073/pnas.1508385112 26240343 PMC4547274

[B20] Holt E. A. Miller S. W. (2011). Bioindicators: using organisms to measure environmental impacts. Nat. Educ. Knowl. 3, 8.

[B21] Hugenholtz P. Goebel B. M. Pace N. R. (1998). Impact of culture-independent studies on the emerging phylogenetic view of bacterial diversity. J. Bacteriol. 180, 4765–4774. 9733676 10.1128/jb.180.18.4765-4774.1998 PMC107498

[B22] Huse S. M. Huber J. A. Morrison H. G. Sogin M. L. Welch D. M. (2007). Accuracy and quality of massively parallel DNA pyrosequencing. Genome Biol. 8, R143. 10.1186/gb-2007-8-7-r143 17659080 PMC2323236

[B23] Jesus E. C. D. Marsh T. L. Tiedje J. M. Moreira F. M. S. (2009). Changes in land use alter the structure of bacterial communities in Western Amazon soils. ISME J. 3, 1004–1011. 10.1038/ismej.2009.47 19440233

[B24] Jones R. T. Robeson M. S. Lauber C. L. Hamady M. Knight R. Fierer N. (2009). A comprehensive survey of soil acidobacterial diversity using pyrosequencing and clone library analyses. ISME J. 3, 442–453. 10.1038/ismej.2008.127 19129864 PMC2997719

[B25] Juo A. S. R. Manu A. (1996). Chemical dynamics in slash-and-burn agriculture. Agric. Ecosyst. Environ. 58, 49–60. 10.1016/0167-8809(95)00656-7

[B26] Jurasinski G. Retzer V. (2012). Simba: A Collection of Functions for Similarity Analysis of Vegetation Data. R package Version 0.3-5. Available online at: http://CRAN.R-project.org/package=simba

[B27] Kielak A. Pijl A. S. van Veen J. A. Kowalchuk G. A. (2009). Phylogenetic diversity of Acidobacteria in a former agricultural soil. ISME J. 3, 378–382. 10.1038/ismej.2008.113 19020558

[B28] Kim J. S. Sparovek G. Longo R. M. De Melo W. J. Crowley D. (2007). Bacteria diversity of terra preta and pristine forest soil from the western Amazon. Soil Biol. Biochem. 39, 684–690. 10.1016/j.soilbio.2006.08.010

[B29] Kim M. Singh D. Lai-Hoe A. Go R. Abdul Rahim R. Ainuddin A. N.. (2012). Distinctive phyllosphere bacterial communities in tropical trees. Microb. Ecol. 63, 674–681. 10.1007/s00248-011-9953-1 21990015

[B30] Klappenbach J. A. Dunbar J. M. Schmidt T. M. (2000). rRNA operon copy number reflects ecological strategies of Bacteria. Appl. Environ. Microbiol. 66, 1328–1333. 10.1128/AEM.66.4.1328-1333.2000 10742207 PMC91988

[B31] Kuramae E. E. Yergeau E. Wong L. C. Pijl A. S. van Veen J. A. Kowalchuk G. A. (2011). Soil characteristics more strongly influence soil bacterial communities than land-use type. FEMS Microbiol. Ecol. 79, 12–24. 10.1111/j.1574-6941.2011.01192.x 22066695

[B32] Lauber C. L. Hamady M. Knight R. Fierer N. (2009). Pyrosequencing-based assessment of soil pH as a predictor of soil bacterial community composition at the continental scale. Appl. Environ. Microbiol. 75, 5111–5120. 10.1128/AEM.00335-09 19502440 PMC2725504

[B33] Lauber C. L. Strickland M. S. Bradford M. A. Fierer N. (2008). The influence of soil properties on the structure of bacterial and fungal communities across land-use types. Soil Biol. Biochem. 40, 2407–2415. 10.1016/j.soilbio.2008.05.021

[B34] Lee S.-H. Cho J.-C. (2011). Group-specific PCR primers for the phylum Acidobacteria designed based on the comparative analysis of 16S rRNA gene sequences. J. Microbiol. Methods 86, 195–203. 10.1016/j.mimet.2011.05.003 21600936

[B35] Li H. Ye D. Wang X. Settles M. L. Wang J. Hao Z. (2014). Soil bacterial communities of different natural forest types in Northeast China. Plant Soil 383, 203–216. 10.1007/s11104-014-2165-y

[B36] Makeschin F. Haubrich F. Abiy M. Burneo J. I. Klinger T. (2008). Pasture management and natural soil regeneration, in Gradients in a Tropical Mountain Ecosystem of Ecuador - Ecological Studies, eds Beck E. Bendix J. Kottke I. Makeschin F. Mosandl R. (Berlin: Springer), 397–408. 10.1007/978-3-540-73526-7_38

[B37] Männistö M. K. Rawat S. Starovoytov V. Häggblom M. M. (2011). Terriglobus saanensis sp. nov., a novel Acidobacterium isolated from tundra soil of Northern Finland. Int. J. Syst. Evol. Microbiol. 61, 1823–1828. 10.1099/ijs.0.026005-0 21186292

[B38] Mirza B. S. Potisap C. Nüsslein K. Bohannan B. J. M. Rodrigues J. L. M. (2014). Response of free-living nitrogen-fixing microorganisms to land use change in the Amazon rainforest. Appl. Environ. Microbiol. 80, 281–288. 10.1128/AEM.02362-13 24162570 PMC3911016

[B39] Mueller R. C. Paula F. S. Mirza B. S. Rodrigues J. L. M. Nüsslein K. Bohannan B. J. M. (2014). Links between plant and fungal communities across a deforestation chronosequence in the Amazon rainforest. ISME J. 8, 1548–1550. 10.1038/ismej.2013.253 24451208 PMC4069395

[B40] Naether A. Foesel B. U. Naegele V. Wüst P. K. Weinert J. Bonkowski M.. (2012). Environmental factors affect acidobacterial communities below the subgroup level in grassland and forest soils. Appl. Environ. Microbiol. 78, 7398–7406. 10.1128/AEM.01325-12 22885760 PMC3457104

[B41] Navarrete A. A. Cannavan F. S. Taketani R. G. Tsai S. M. (2010). A molecular survey of the diversity of microbial communities in different Amazonian agricultural model systems. Diversity 2, 787–809. 10.3390/d2050787

[B42] Navarrete A. A. Kuramae E. E. de Hollander M. Pijl A. S. van Veen J. A. Tsai S. M. (2013). Acidobacterial community responses to agricultural management of soybean in Amazon forest soils. FEMS Microbiol. Ecol. 83, 607–621. 10.1111/1574-6941.12018 23013447

[B43] Navarrete A. A. Taketani R. G. Mendes L. W. Cannavan F. S. Moreira F. M. S. Tsai S. M. (2011). Land-use systems affect archaeal community structure and functional diversity in western Amazon soils. Rev. Bras. de Cienc. Solo 35, 1527–1540. 10.1590/s0100-06832011000500007

[B44] Navarrete A. A. Tsai S. M. Mendes L. W. Faust K. de Hollander M. Cassman N.. (2015). Soil microbiome responses to the short-term effects of Amazonian deforestation. Mol. Ecol. 24, 2433–2448. 10.1111/mec.13172 25809788

[B45] Neill C. Fry B. Melillo J. M. Steudler P. A. Moraes J. F. L. Cerri C. C. (1996). Forest and pasture derived carbon contributions to carbon stocks and microbial respiration of tropical pasture soils. Oecologia 107, 113–119. 10.1007/BF00582241 28307198

[B46] Neill C. Piccolo M. C. Steudler P. A. Melillo J. M. Feigl B. J. Cerri C. C. (1995). Nitrogen dynamics in soils of forests and active pastures in the western Brazilian Amazon basin. Soil Biol. Biochem. 27, 1167–1175. 10.1016/0038-0717(95)00036-E

[B47] Neye P. Greenland D. (1960). The soil under shifting cultivation. Tech. Commun. Common Wealth Agric. Bur. 51, 156.

[B48] Noll M. Matthies D. Frenzel P. Derakshani M. Liesack W. (2005). Succession of bacterial community structure and diversity in a paddy soil oxygen gradient. Environ. Microbiol. 7, 382–395. 10.1111/j.1462-2920.2005.00700.x 15683399

[B49] Olden J. D. Poff N. L. (2003). Toward a mechanistic understanding and prediction of biotic homogenization. Am. Nat. 162, 442–460. 10.1086/378212 14582007

[B50] Paula F. S. Rodrigues J. L. M. Zhou J. Wu L. Mueller R. C. Mirza B. S.. (2014). Land use change alters functional gene diversity, composition and abundance in Amazon forest soil microbial communities. Mol. Ecol. 23, 2988–2999. 10.1111/mec.12786 24806276

[B51] Pollard K. S. Dudoit S. van der Laan M. J. (2005). “Multiple testing procedures: R multtest package and applications to genomics” in Bioinformatics and computational biology solutions using R and bioconductor, eds Gentleman R. Carey V. Dudoit S. Irizarry R. Huber W. (New York, NY: Springer), 251–272.

[B52] Potthast K. Hamer U. Makeschin F. (2010). Impact of litter quality on mineralization processes in managed and abandoned pasture soils in Southern Ecuador. Soil Biol. Biochem. 42, 56–64. 10.1016/j.soilbio.2009.09.025

[B53] Ranjan K. Paula F. S. Mueller R. C. Jesus E. C. Cenciani K. Bohannan B. J.. (2015). Forest-to-pasture conversion increases the diversity of the phylum Verrucomicrobia in Amazon rainforest soils. Front. Microbiol. 6:779. 10.3389/fmicb.2015.00779 26284056 PMC4519759

[B54] Rawat S. R. Männistö M. K. Bromberg Y. Haggblom M. M. (2012). Comparative genomic and physiological analysis provides insights into the role of *Acidobacteria* in organic carbon utilization in Arctic tundra soils. FEMS Microbiol. Ecol. 82, 341–355. 10.1111/j.1574-6941.2012.01381.x 22486608

[B55] Rawat S. R. Männistö M. K. Starovoytov V. Goodwin L. Nolan M. Hauser L.. (2014). Complete genome sequence of *Granulicella tundricola* type strain MP5ACTX9T, an *Acidobacteria* from tundra soil. Stan. Genomic Sci. 9, 449–461. 10.4056/sigs.4648353 25197431 PMC4148992

[B56] Rawat S. R. Männistö M. K. Starovoytov V. Goodwin L. Nolan M. Hauser L. J.. (2013). Complete genome sequence of *Granulicella mallensis* type strain MP5ACTX8T, an *acidobacterium* from tundra soil. Stand. Genomic Sci. 9, 71–82. 10.4056/sigs.4328031 24501646 PMC3910553

[B57] R Core Team (2015). R: A Language and Environment for Statistical Computing. R Foundation for Statistical Computing. Vienna. Available online at: http://www.R-project.org/

[B58] Rhoades C. C. Coleman D. C. (1999). Nitrogen mineralization and nitrification following land conversion in montane Ecuador. Soil Biol. Biochem. 31, 1347–1354. 10.1016/S0038-0717(99)00037-1

[B59] Rhoades C. C. Eckert G. E. Coleman D. C. (2000). Soil carbon differences among forest, agriculture, and secondary vegetation in lower montane Ecuador. Ecol. Appl. 10, 497–505. 10.1890/1051-0761(2000)010[0497:SCDAFA]2.0.CO;2

[B60] Rodrigues J. L. M. Pellizari V. H. Mueller R. Baek K. Jesus E. C. Paula F. S.. (2013). Conversion of the Amazon rainforest to agriculture results in biotic homogenization of soil bacterial communities. Proc. Natl. Acad. Sci. U.S.A. 110, 988–993. 10.1073/pnas.1220608110 23271810 PMC3549139

[B61] Rousk J. Bååth E. Brookes P. C. Lauber C. L. Lozupone C. Caporaso J. G.. (2010). Soil bacterial and fungal communities across a pH gradient in an arable soil. ISME J. 4, 1340–1351. 10.1038/ismej.2010.58 20445636

[B62] Sait M. Davis K. E. Janssen P. H. (2006). Effect of pH on isolation and distribution of members of subdivision 1 of the phylum *Acidobacteria* occurring in soil. Appl. Environ. Microbiol. 72, 1852–1857. 10.1128/AEM.72.3.1852-1857.2006 16517631 PMC1393200

[B63] Sala O. E. Chapin F. S. Armesto J. J. Berlow E. Bloomfield J. Dirzo R.. (2000). Biodiversity - global biodiversity scenarios for the year 2100. Science 287, 1770–1774. 10.1126/science.287.5459.1770 10710299

[B64] Stevenson B. S. Schmidt T. M. (2004). Life history implications of rRNA gene copy number in *Escherichia coli*. Appl. Environ. Microbiol. 70, 6670–6677. 10.1128/AEM.70.11.6670-6677.2004 15528533 PMC525164

[B65] Taketani R. G. Tsai S. M. (2010). The influence of different land uses on the structure of archaeal communities in Amazonian anthrosols based on 16S rRNA and *amo*A genes. Microb. Ecol. 59, 734–743. 10.1007/s00248-010-9638-1 20204349

[B66] Wang Q. Garrity G. M. Tiedje J. M. Cole J. R. (2007). Naive Bayesian classifier for rapid assignment of rRNA sequences into the new bacterial taxonomy. Appl. Environ. Microbiol. 73, 5261–5267. 10.1128/AEM.00062-07 17586664 PMC1950982

[B67] Ward N. L. Challacombe J. F. Janssen P. H. Henrissat B. Coutinho P. M. Wu M.. (2009). Three genomes from the phylum *Acidobacteria* provide insight into the lifestyles of these microorganisms in soils. Appl. Environ. Microb. 75, 2046–2056. 10.1128/AEM.02294-08 19201974 PMC2663196

[B68] Wearn O. R. Reuman D. C. Ewers R. M. (2012). Extinction debt and windows of conservation opportunity in the Brazilian Amazon. Science 337, 228–232. 10.1126/science.1219013 22798612

[B69] Zhao J. Ni T. Li Y. Xiong W. Ran W. Shen B.. (2014). Responses of bacterial communities in arable soils in a rice-wheat cropping system to different fertilizer regimes and sampling times. PLoS ONE 9:e85301. 10.1371/journal.pone.0085301 24465530 PMC3896389

[B70] Zimmermann J. Gonzalez J. M. Saiz-Jimenez C. Ludwig W. (2005). Detection and phylogenetic relationships of highly diverse uncultured acidobacterial communities in altamira cave using 23S rRNA sequence analyses. Geomicrobiol. J. 22, 379–388. 10.1080/01490450500248986

