# Supplementary material for: Differential Response of Acidobacteria Subgroups to Forest-to-Pasture Conversion and Their Biogeographic Patterns in the Western Brazilian Amazon
Source: Front Microbiol. 2015 Dec 22;6:1443. doi: 10.3389/fmicb.2015.01443 (PMC4686610; doi:10.3389/fmicb.2015.01443)
Supplement: Supplementary file 1 [file Table1.PDF]

Table S1. List of samples and nucleotide barcode sequence

ID Sample	Nucleotide barcode sequence
F1_A1	TGCATACACTGG
F1_A10	AGTCGAACGAGG
F1_A100	ACCAGTGACTCA
F1_B1	GAATACCAAGTC
F1_B10	GTAGATCGTGTA
F1_B100	TAACGTGTGTGC
F1_C1	CATTATGGCGTG
F1_C10	CCAATACGCCTG
F1_C100	GATCTGCGATCC
F2_A1	GCAACACCATCC
F2_A10	GCGATATATCGC
F2_A100	CGAGCAATCCTA
F2_B1	CGAGGGAAAGTC
F2_B10	CAAATTCGGGAT
F2_B100	AGTTGAGGCATT
F2_C1	ACAATAGACACC
F2_C10	AGTTACGAGCTA
F2_C100	GCATATGCACTG
F3_A1	AGTCGTGCACAT
F3_A10	GTATCTGCGCGT
F3_A100	TTGCGTTAGCAG
F3_B1	TACGAGCCCTAA
F3_B10	CGGTCAATTGAC
F3_B100	GTGGAGTCTCAT
F3_C1	TGCAGTCCTCGA
F3_C10	ACCATAGCTCCG
F3_C100	TCGACATCTCTT
P1_A1	TTGGCTCTATTC
P1_A10	GATCCCACGTAC
P1_A100	TACCGCTTCTTC
P1_B1	TGTGCGATAACA
P1_B10	GATTATCGACGA
P1_B100	GCCTAGCCCAAT
P1_C1	ACTCCTTGTGTT
P1_C10	GTCACGGACATT
P1_C100	GCGAGCGAAGTA
P2_A1	TCTTGGAGGTCA
P2_A10	TCACCTCCTTGT
P2_A100	GCACACCTGATA
P2_B1	GCGACAATTACA
P2_B10	TCATGCTCCATT
P2_B100	AGCTGTCAAGCT
P2_C1	GAGAGCAACAGA
P2_C10	TACTCGGGAACT
P2_C100	CGTGCTTAGGCT
P3_A1	GTATTTCGGACG
P3_A10	TATCTATCCTGC
P3_A100	TTGCCAAGAGTC
P3_B1	AGTAGCGGAAGA
P3_B10	GCAATTAGGTAC
P3_B100	CATACCGTGAGT
P3_C1	CCTGCGAAGTAT
P3_C10	TTCTCTCGACAT
P3_C100	GCTCTCCGTAGA
