# Supplementary material for: Differential Response of Acidobacteria Subgroups to Forest-to-Pasture Conversion and Their Biogeographic Patterns in the Western Brazilian Amazon
Source: Front Microbiol. 2015 Dec 22;6:1443. doi: 10.3389/fmicb.2015.01443 (PMC4686610; doi:10.3389/fmicb.2015.01443)
Supplement: Supplementary file 2 [file Table2.PDF]

Table S2. Soil chemical properties of the 0 to 10 cm topsoil layer at different sampling sites

Soil properties	Forest sites			Pasture sites			Statistics
	F1	F2	F3	P1	P2	P3	F *vs.* P
pH	5.02[1]±0.5[2]	5.25±0.6	4.75±0.3	4.97±0.2	4.91±0.1	5.26±0.3	ns[3]
N	0.09±0	0.13±0	0.06±0	0.13±0	0.13±0	0.17±0	***
C	1.16±0.2	1.52±0.6	0.69±0.3	1.79±0.6	1.75±0.2	2.06±0.2	***
C/N	12.20±1.2	11.93±1.7	12.85±1.1	13.31±1.7	13.53±0.9	12.26±0.5	*
P	7.89±1.4	10.56±4.8	5.56±2.1	6.78±1.9	5.89±0.9	14.78±5.1	ns
S	4.56±1.3	3.89±1.3	3.33±0.5	4.56±0.5	4.89±1.1	5.22±0.7	**
K	2.51±2.5	1.96±1.0	1.14±0.7	1.62±0.7	1.46±0.7	2.32±0.9	ns
Ca	18.44±7.9	34.33±22.4	7.44±4.0	15.0±7.1	11.33±2.2	18.22±2.3	ns
Mg	7.0±1.7	6.56±2.2	4.0±1.7	7.11±1.5	5.67±1.3	8.89±2.9	*
Al	0.78±0.7	0.44±1.1	2.33±1.1	1.0±0	1.22±0.4	1.11±0.3	ns
H+Al	27.56±4.4	27.67±4.4	25.44±10.7	32.0±5.1	36.78±3.8	33.67±4.5	***
CEC	55.51±8.2	70.51±21.0	38.03±13.7	55.73±12.3	55.23±4.2	63.10±2.7	ns
V	48.89±12.5	56.78±16.6	32.67±12.3	41.67±6.6	33.44±4.9	46.56±7.2	ns
m	3.56±2.1	2.78±4.5	18.78±11.5	4.56±1.3	6.56±3.2	3.89±1.6	ns

Ca, Mg, K, Al, potential acidity (H+Al), cation exchange capacity in pH 7 (CEC) are expressed in mmolc$_c$ dm^{-3}; P is expressed in mg dm^{-3}; Al saturation index (m). Base saturation index (V).

(1) The values are averages based on nine replicate sampling points in each site

(2) Standard deviation of the average of nine soil replicates

(3) Tukey's honestly significant difference (HSD) test was performed considering forest *vs.* pasture including all sampling sites across 27 soil cores for forest sites and 27 soil cores for pasture sites. Significance levels: ns: *P*>0.05, **P*<0.05, ***P*<0.005, ****P*<0.0005
