# Supplementary material for: Differential Response of Acidobacteria Subgroups to Forest-to-Pasture Conversion and Their Biogeographic Patterns in the Western Brazilian Amazon
Source: Front Microbiol. 2015 Dec 22;6:1443. doi: 10.3389/fmicb.2015.01443 (PMC4686610; doi:10.3389/fmicb.2015.01443)
Supplement: Supplementary file 3 [file Table3.PDF]

Table S3. Size in base pair and number of bacterial and acidobacterial sequence reads obtained by barcoded pyrosequencing for each site

	Size (bp[(1)])	Number of sequence reads matching the bacterial 16S rRNA gene[(2)]	Number of sequence reads matching the acidobacterial 16S rRNA gene[(2)]
Forest sites			
F1	330 ± 6[(3)]	4735 ± 278	973 (20.5%) ± 33 (3.4%)[(4)]
F2	330 ± 4	4632 ± 215	934 (20.2%) ± 32 (3.4%)
F3	332 ± 6	4596 ± 253	940 (20.4%) ± 34 (3.6%)
Pasture sites			
P1	330 ± 8	5120 ± 354	536 (10.5%) ± 18 (3.3%)
P2	332 ± 4	5247 ± 326	586 (11.2%) ± 19 (3.2%)
P3	330 ± 6	5417 ± 309	610 (11.3%) ± 21 (3.4%)

(1) Post quality control.

(2) Sequences taxonomy assignments were made using Ribosomal Database Project (RDP) 2.6 classifier.

(3) Average and range of the average for each nine replicate soil sample in each site.

(4) Average percentage and range of the average of acidobacterial sequences across the three individual samples for each site. The percentual value was calculated by comparing the number of sequences classified as belonging to *Acidobacteria* vs. the number of classified bacterial sequences.
