# Supplementary material for: Differential Response of Acidobacteria Subgroups to Forest-to-Pasture Conversion and Their Biogeographic Patterns in the Western Brazilian Amazon
Source: Front Microbiol. 2015 Dec 22;6:1443. doi: 10.3389/fmicb.2015.01443 (PMC4686610; doi:10.3389/fmicb.2015.01443)
Supplement: Supplementary file 4 [file Image1.pdf]

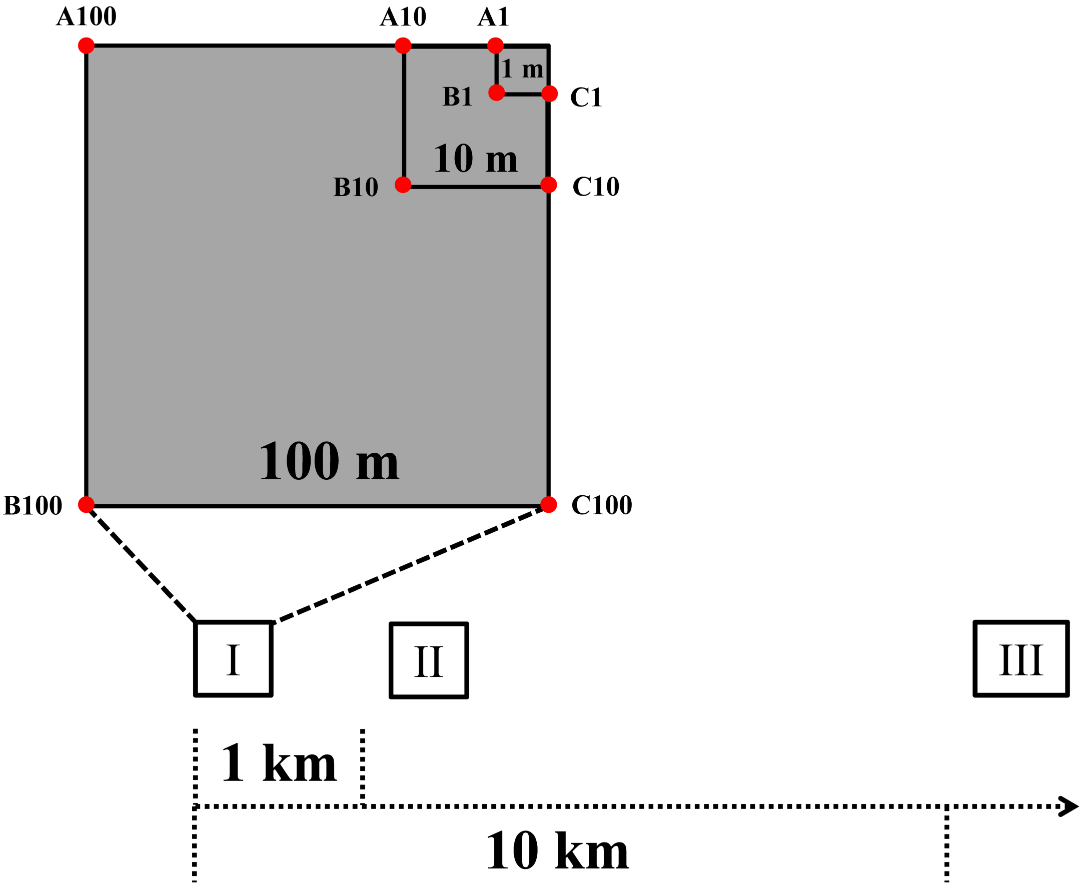

Figure S1. Sampling scheme. Three one-hectare plots (denoted I, II, and III in the diagram) were established in both pasture and forest, spaced at 1-km and 10-km distances. Nested 10-m^2 and 1-m^2 plots were established within each hectare quadrat (depicted in the detail blowup of the diagram). Soil cores (red points in the diagram) were taken at the corners of each plot at each scale (for a total of nine cores per hectare plot, and 36 per 10-km transect).
