# Supplementary material for: Differential Response of Acidobacteria Subgroups to Forest-to-Pasture Conversion and Their Biogeographic Patterns in the Western Brazilian Amazon
Source: Front Microbiol. 2015 Dec 22;6:1443. doi: 10.3389/fmicb.2015.01443 (PMC4686610; doi:10.3389/fmicb.2015.01443)
Supplement: Supplementary file 5 [file Image2.pdf]

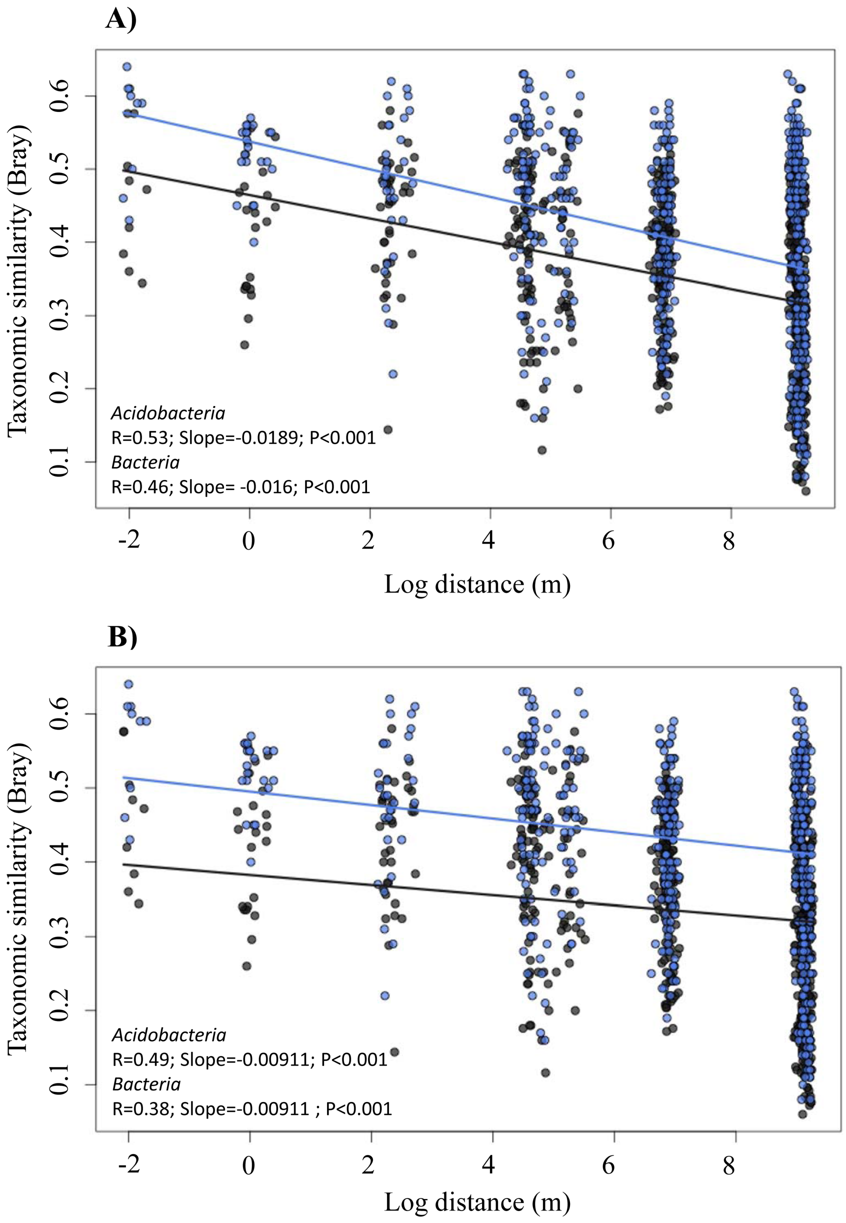

Figure S2. Decay of taxonomic similarity (Bray–Curtis) with geographic distance in forest (A) and pasture (B) for *Acidobacteria* at the phylum level (blue) and for total *Bacteria* (dark gray).
